# TGFβRI antagonist inhibits HIV-1 Nef-induced CC chemokine family ligand 2 (CCL2) in the brain and prevents spatial learning impairment

**DOI:** 10.1186/s12974-019-1664-4

**Published:** 2019-12-11

**Authors:** Gladys Chompre, Neysha Martinez-Orengo, Myrella Cruz, James T. Porter, Richard J. Noel

**Affiliations:** 1 Biology Department, Pontifical Catholic University of Puerto Rico, Ponce, Puerto Rico; 2 Department of Basic Sciences, Ponce Health Sciences University-Ponce Medical School, Ponce Research Institute, P.O. Box 7004, Ponce, PR 00731 USA

**Keywords:** Hippocampus, HAND, Cognition, TGFβ-1, CCL2, Inflammation

## Abstract

**Background:**

HIV-1–associated neurocognitive disorders (HAND) progression is related to continued inflammation despite undetectable viral loads and may be caused by early viral proteins expressed by latently infected cells. Astrocytes represent an HIV reservoir in the brain where the early viral neurotoxin negative factor (Nef) is produced. We previously demonstrated that astrocytic expression of Nef in the hippocampus of rats causes inflammation, macrophage infiltration, and memory impairment. Since these processes are affected by TGFβ signaling pathways, and TGFβ-1 is found at higher levels in the central nervous system of HIV-1+ individuals and is released by astrocytes, we hypothesized a role for TGFβ-1 in our model of Nef neurotoxicity.

**Methods:**

To test this hypothesis, we compared cytokine gene expression by cultured astrocytes expressing Nef or green fluorescent protein. To determine the role of Nef and a TGFβRI inhibitor on memory and learning, we infused astrocytes expressing Nef into the hippocampus of rats and then treated them daily with an oral dose of SD208 (10 mg/kg) or placebo for 7 days. During this time, locomotor activity was recorded in an open field and spatial learning tested in the novel location recognition paradigm. Postmortem tissue analyses of inflammatory and signaling molecules were conducted using immunohistochemistry and immunofluorescence.

**Results:**

TGFβ-1 was induced in cultures expressing Nef at 24 h followed by CCL2 induction which was prevented by blocking TGFβRI with SD208 (competitive inhibitor). Interestingly, Nef seems to change the TGFβRI localization as suggested by the distribution of the immunoreactivity. Nef caused a deficit in spatial learning that was recovered upon co-administration of SD208. Brain tissue from Nef-treated rats given SD208 showed reduced CCL2, phospho-SMAD2, cluster of differentiation 163 (CD163), and GFAP immunoreactivity compared to the placebo group.

**Conclusions:**

Consistent with our previous findings, rats treated with Nef showed deficits in spatial learning and memory in the novel location recognition task. In contrast, rats treated with Nef + SD208 showed better spatial learning suggesting that Nef disrupts memory formation in a TGFβ-1-dependent manner. The TGFβRI inhibitor further reduced the induction of inflammation by Nef which was concomitant with decreased TGFβ signaling. Our findings suggest that TGFβ-1 signaling is an intriguing target to reduce neuroHIV.

## Background

Combination antiretroviral therapy (cART) significantly prolongs the lifespan of individuals infected with HIV-1. Despite this advancement, new cases of HIV-1-associated neurocognitive disorders (HAND) continue to develop. HAND is subclassified into HIV-associated dementia (HAD), mild neurocognitive disorder (MND), and asymptomatic neurocognitive impairment (ANI), with the latter two currently affecting approximately 50% of the HIV-1-positive individuals [1], including those on cART. Individuals living with HIV-1 are seven times more susceptible to develop mild cognitive impairment when compared to HIV negative counterparts [2]. This is a concern since mild cognitive impairment typically precedes dementia [3]. The production of viral proteins from latently infected cells in the brain, regardless of the use of cART, contributes to these impairments [4].

Damage due to viral neurotoxic proteins in the hippocampus adversely affects memory formation and causes HIV-related cognitive impairment [5]. Hippocampal neuronal death can result from the inflammatory action of cytokines and viral neurotoxins including negative factor (Nef) [6]. Nef sequences were also found in microdissected human hippocampus tissue from HIV+ patients, presenting the brain as a potential viral reservoir upon virus reactivation [7]. Furthermore, significant amounts of Nef protein were identified in astrocytes from postmortem brain tissues of HIV-infected humans and the brains of macaques with SIV encephalitis [8, 9].

The continuous production of HIV-1 Nef by monocytes, macrophages, and astrocytes induces the secretion of inflammatory cytokines, perpetuating an inflammatory condition that creates a cytotoxic environment for cells in the brain and periphery [10–14]. We previously found in our animal model that Nef increases CC chemokine family ligand 2 (CCL2) and infiltration of macrophages into the hippocampus after infusion of astrocytes expressing Nef [15]. Neuronal death can be triggered by the overproduction of cytokines, such as CCL2, interferon gamma-induced protein-10 (CXCL10/IP-10), and transforming growth factor beta-1 (TGFβ-1) [6, 16]. The endogenous production of Nef is enough to induce an immune response which turns on the crosstalk between different cell types and signaling pathways [17] suggesting a mechanism for Nef-mediated neurotoxicity in our model.

The TGFβ signaling pathway exerts a number of contrasting effects in cells (e.g., B cells, CD4+ T cells, dendritic cells, monocytes, astrocytes) which have also been described in relation with HIV infection; these effects include changes in proliferation [18], differentiation [19], apoptosis [20], and chemotaxis [21]. Although TGFβ-1 can be neuroprotective [22, 23], other studies suggest that an overexpression of TGFβ-1 causes neurodegeneration [24]. HIV infection and neurological disorders such as Alzheimer’s disease show an increased expression of TGFβ-1 and its receptor [25, 26]. TGFβ-1 is produced by astrocytes and microglia after neuronal injury [27] while hippocampal neurons express a high concentration of the TGFβRI [28]. In addition, TGFβ-1 enhances glutamatergic currents in hippocampal neurons which could lead to neurotoxicity [29]. Overexpression of TGFβ-1 in the hippocampus produces inhibition of neurogenesis [30] suggesting a wide range of possible involvement of TGFβ-1 signaling in our model. Following our published findings that astrocytes expressing Nef increase inflammation leading to impaired memory when compared to GFP controls, we consider TGFβ-1 signaling a significant mechanism for investigation in the context of HIV-1 neuropathologies.

We examined the role of TGFβ-1 signaling using a pharmacological approach to interrupt the Nef-induced spatial memory impairment. SD208 is a competitive and specific inhibitor for TGFβRI kinase which blocks autocrine and paracrine TGFβ signaling [31] by preventing the phosphorylation of the SMADs [32]. This TGFβRI inhibitor is a small molecule that crosses the blood brain barrier upon systemic administration [33]. It can prevent neurological deficits in a dose-dependent manner when administered intraperitoneally as shown with approaches targeting its effect on the brain via immunoreactivity and behavioral studies [33]. Here, we report that astrocytes expressing Nef induce TGFβ and CCL2, cause inflammation shown by increased cluster of differentiation 163 (CD163) and glial fibrillary acidic protein (GFAP) immunoreactivity, and activate the TGFβ signaling pathway as demonstrated with the increased phosphorylation of SMAD2. These signaling and inflammatory changes are prevented by co-administration of SD208 with which learning and memory are returned to normal.

## Methods

### Ethics statement

All animal experiments were conducted in accordance with the Guide for the Care and Use of Laboratory Animals and with the approval of the Institutional Animal Care and Use Committee from Ponce Health Sciences University.

### Culture of primary astrocytes

Primary astrocytes were extracted following the procedure described previously [15, 34]. The cells were extracted from 2- to 3-month-old Sprague Dawley rats. Brains were removed following deep anesthesia with halothane and decapitation. Brain tissue was minced with scissors in Hank’s balanced salt solution (Sigma, St. Louis, MO) on ice. The minced brain was transferred to a tube with trypsin and incubated at 37 °C for 10 min. Trypsin was inactivated using Dulbecco’s modified Eagle media (DMEM) supplemented with 10% fetal bovine serum, and larger pieces of tissue were separated from suspended cells by sedimentation. The process was performed repeatedly on the larger tissue pieces, and all cells in suspension were combined, washed, and seeded in complete medium. After 3 days, the culture media was changed to remove non-attached cells.

Purity of astrocyte cultures was assessed using immunofluorescence staining and western blot for GFAP (astrocyte marker, positive) and Iba1/2 (presence of microglia, negative) [15]. Neurons and oligodendrocytes do not survive under these culture conditions; microglia do not attach to the plate and are removed in the first media change. As a result, near 100% astrocyte purity was inferred since other cells remain undetectable. The astrocytes survived and continued to express Nef in vitro for at least 7 days. In vivo, we detected Nef by immunofluorescence and immunohistochemistry at the infusion site at the time of sacrifice, demonstrated at 7 days [15] and 10 days [35].

### Transient transfection of primary astrocytes

Astrocytes were transfected with a plasmid containing the Nef gene under control of the CMV immediate early promoter obtained from NIH AIDS Reference Research and Reagent Program (Cat. # 8677) or a GFP gene plasmid as a control. The Nef plasmid encodes the full 206 amino acid protein, is codon optimized for human expression, and contains 2 of the 5 consensus amino acids for brain derived variants [36]. For transfections, 1.6 million cells were mixed with 5 μg of Nef or GFP plasmid and pulsed for 35 ms at 250 V in 4-mm cuvettes using a BioRad Gene Pulser Xcell electroporator. Immunoblotting was performed to confirm Nef (Nef EH1 antibody 1:1000 dilution, #3689 provided by the NIH AIDS Reagent Program) or GFP (1:5000 dilution, Abcam #6556) protein expression. To test the expression of TGFβ-1 and CCL2 in vitro, 3.2 million transfected cells were grown in a 25-cm^2^ flask. We also confirmed the expression of the TGFβRI in the transfected cells by seeding 20,000 cells/well in two-well chambers. Cells were fixed after 24 h, permeabilized with 0.5% Triton, blocked with 2% BSA, and incubated with the primary antibody (TGFβRI 1:500 dilution Abcam #31013) followed by incubation with secondary antibody (1:1000 dilution Alexa Fluor 555). For in vivo experiments, transfected cells were adjusted to 200,000 cells/μL and infusions were performed using 0.5 μL to deliver 100,000 cells into the right hippocampus.

### qPCR of TGFβ-1 and CCL2 messenger RNA

Primary astrocytes were transfected with the Nef or GFP plasmids and incubated with or without the inhibitor, SD208 from Tocris (5 μM, daily). Cells were collected at different time points (24 and 48 h). Extraction of RNA was done using the All Prep kit (Qiagen, Valencia, CA), and the quality of the RNA was verified and quantified using an Experion Automated electrophoretic workstation (BioRad). RNA was converted to cDNA using iScript cDNA synthesis kit from BioRad. TGFβ-1 and CCL2 amplification was performed using SYBR Green Super mix from BioRad. Primers were obtained from Qiagen. Beta actin amplification was used as an internal control. Relative expression was determined using the 2^−ΔΔC^_T_ method normalized using beta actin. We reported the relative expression compared to the GFP group.

### Total SMAD4 immunoassay

For this experiment, astrocytes and cortical neurons were obtained from E18 Sprague Dawley rats. Tissue homogenates were filtered and separated into two groups to obtain the two types of cells. Astrocytes were isolated and cultured as previously described. To obtain cortical neurons, a total of 4.0 × 10^4^ cells were seeded in 12-well plates in 2 ml/well of DMEM-F12. Cells were incubated with 5% CO_2_ at 37 °C. After 24 h, the medium was removed and substituted with 2 ml/well of neurobasal medium (without FBS). Every week, 1 ml/well of medium was removed and refreshed with 1 ml/well neurobasal medium. Cortical neurons were ready to use for experiments between the second and third week after isolation. The day of the experiment (2–3 weeks after isolation of cortical neurons), a total of 4.0 × 10^4^ transfected rat primary astrocytes were seeded on inserts and placed on the respective wells containing cortical neurons. Another group of transfected cells in the transwell co-culture system was treated with 5 μM SD208 which was refreshed every 24 h. Astrocytes and cortical neuron lysates were collected at 24, 48, and 72 h and assayed with a magnetic bead kit (Millipore cat. # 48-614MAG) following the manufacturer’s protocol. Median fluorescent intensity (MFI) was obtained from the Luminex system.

### Animals for surgery and behavioral studies

Thirty-day-old, male Sprague Dawley rats were used to test SD208 efficacy in preventing Nef learning impairment in the novel location recognition (NLR) task. Rats were housed in a controlled-access room with a 12-h light/dark interval with free access to food and water and were weighed each day. There were two groups in this study: rats with implanted astrocytes expressing Nef received a daily oral dose of either a placebo/vehicle (1% methylcellulose dissolved in distilled water) (*n* = 7) or SD208 dissolved in 1% methylcellulose (10 mg/kg) (*n* = 13). After surgery and recovery, the rats received daily oral treatment with placebo or SD208 (10 mg/kg). Rats were caged in groups of two or three to avoid isolation stress. Procedures were approved by the Institutional Animal Care and Use Committee.

### Infusion of astrocytes expressing Nef

Infusions of astrocytes were performed following our previous protocol with minor modifications [15, 34, 37]. The rats were placed in an acrylic box and anesthesia induced with 5% isoflurane. After induction, the rats were maintained under anesthesia with 1.5–2.0% of isoflurane. Astrocytes expressing Nef were infused in the right hippocampal hemisphere using the following coordinates: anterior posterior − 0.28 mm, midlateral ± 0.17 mm, and dorsoventral − 0.37 mm. The 100,000 transfected cells (in a volume of 0.5 μL) were administered at a constant rate over one minute. The needle was left in place for five minutes following infusion to prevent backflow along the needle track.

### Application of the TGFβ receptor I inhibitor (SD208)

SD208 was administered orally (10 mg/kg) once daily after surgery until sacrifice. The placebo group (control) was infused with astrocytes expressing Nef and received daily oral doses of the vehicle (1% methylcellulose).

### Novel location recognition

Novel location recognition (NLR) was performed using our previously validated protocol in which the implantation of astrocytes expressing green fluorescent protein (GFP) in the rat hippocampus showed the same learning performance as a naive animal in novel location and novel object recognition [15]. Following surgery, rats recovered for 2 days in a clean cage. On the third, fourth, and fifth days post-surgery, the animals were habituated in an empty box (36 × 36 inches) for 5 min per day. The box was located in a room (7.5 × 7.5 feet) with fixed spatial cues on the walls and a video camera above the testing chamber. The video data was collected and analyzed using Ethovision software which can detect three body parts of the rat (nose, center, and tail). The nose point was used to measure the exploration time, and the center point was used to measure the mobility of the rats. On the sixth day, unique objects were placed in three of the four corners of the box for the learning phase. Each animal was placed in the box for three trials of 10-min each at 1-h intervals. Between each cycle, the box and objects were cleaned with 70% ethanol to eliminate odor cues. After the three trials, all animals were returned to their cages for a 24-h period. The following day (day 7), one of the three objects was relocated to the previously empty corner (new location). Each animal was given a single trial of 10 min to test learning. Learning was indicated by the preference to explore the object in the novel location, whereas failure to learn was indicated by lack of preference for the moved object.

### Immunohistochemistry of phospho-SMAD2, CD163, and GFAP

After performing the novel location test (on day 7), the animals were anesthetized with an overdose of pentobarbital and cardiac puncture/exsanguination commenced upon induction of deep anesthesia. The animals were perfused with saline followed by 10% formaldehyde. The brains were removed and stored in 10% formaldehyde in a histological cassette. Tissues were dehydrated using ascending grades of ethanol (70, 80, 90, 95, 100%) for an hour each and then incubated twice with methyl salicylate for 30 min each. The tissues were left overnight in an incubator at 57 °C in a solution of methyl salicylate and paraffin (1:1). Brain tissues were embedded in paraffin, cut at 8 μm using a microtome and placed onto positive charged glass slides for immunohistochemistry. The mounted tissue slices were deparaffinized in xylene for 30 min and then hydrated in descending grades of ethanol (100, 95, 80, and 70%) to distilled water for 3 min each. Slides were washed with 3% hydrogen peroxide for 15 min to eliminate the endogenous peroxidase from the sample prior to a single wash with phosphate-buffered saline (PBS) and incubation for 45 min in 0.01 M Citrate-EDTA buffer, pH = 6.2 at 90–95 °C for antigen retrieval. After the incubation, the slides were left at room temperature for 20 min followed by a wash with PBS for 5 min. Tissues were covered with protein block (Biogenex, Co.) for 15 min in a humidified chamber to prevent non-specific binding of primary antibodies. The primary antibodies (phospho-SMAD2 (1:10) from Millipore, CD163 (1:10) from Cell Sciences, and GFAP (1:50, from BioLegend) were incubated overnight in a humidified chamber at 4 °C. A negative control tissue was used by adding PBS instead of primary antibody to verify specificity of detection. Slides were washed once with PBS followed by the incubation of a multilink secondary antibody for 20 min in a humidified chamber. Slides were washed with PBS followed by incubation with streptavidin-HRP for 20 min (Super Sensitive Link-Label IHC Detection System, Biogenex Co.; Cat. No.LP000-ULE). Tissues were washed with PBS and developed using a drop of 3,3′ diaminobenzidine (DAB) solution (Cat. # HK153, Biogenex Co.) for 1 min. The reaction of DAB solution was stopped by submersion of the slide in distilled water. Then, tissues were counterstained with hematoxylin (1:2) for 15 s followed by a rinse in running water for 5 min. The slides were dehydrated through ascending graded ethanol (70, 80, 95, 100%) for 2 min each and then cleared in xylene for 2 min. Tissues were mounted using cytoseal XYL (Cat. # 8312-4 Richard Allan Scientific). Photos of brain tissues were taken using an Olympus microscope under the same exposure parameters and high-quality resolution (2460 × 1920 pixels). Densitometry was performed using NIS-elements software from Nikon to determine if there was a statistical change in staining intensity between the control and the experimental group taking into consideration the percent of DAB from each photo and subtracting the background.

### CCL2 immunofluorescence

Mid brain sections were cut at 8 μm using a microtome and placed on a positively charged slide. Slides were deparaffinized in xylene followed by hydration in descending grade of ethanol (100% to 70%), for 3 minutes each. Antigen retrieval incubation was made with 0.01 M Citrate-EDTA buffer (pH = 6.2) at 90–95 °C for 40 min. Non-specific binding was prevented by adding protein block (Biogenex, Co.) on the tissue for 15 min. Tissues with primary CCL2 antibody (1:500) from Millipore were incubated overnight in a humidified chamber at 4 °C. A negative control was included by adding PBS instead of primary antibody. On the second day, slides were washed twice with PBS and the secondary antibody (1:100) (488 Alexa Fluor mouse Life Technologies #A11029) was added and incubated for 30 min in the dark. This was followed by two PBS washes of 5 min each and incubation with DAPI for 5 min to label the nuclei. Tissues were washed twice with PBS, and a drop of antibleaching mounting media (Fluorogel Electron Microscopy Sciences) was added to place the coverslip. Three representative images per brain region (DG, CA1, CA2, CA3) were taken using an Olympus microscope. ImageJ software was used to measure the mean fluorescence intensity.

### Rigor and statistical analysis

Animal experiments, both live behavioral and post-sacrifice tissue analyses, were conducted to allow data collection and initial analysis by blinded observers. The experimental treatments were coded, and animal groupings were unmasked after data were collected and preliminary analyses conducted. Unmasking permitted statistical analyses and interpretation of results using an unbiased data set.

NIS-elements and Graph Pad Prism version 7.0 were used to perform the statistics. Non-parametric unpaired Student’s *T* test was used to measure the differences in quantitative PCR, immunostaining, immunohistochemistry, and rat behavior (mobility of the rats, anxiety levels, velocity, preferences in the learning phase and testing phase). ANOVA was used to assess the preference of the object in the learning phase and the expression of total SMAD4 in vitro (followed by Bonferroni correction). *P* values less than or equal to 0.05 were considered significant. Values were expressed using standard error of the mean.

## Results

### Nef increases the expression of TGFβ-1 in astrocytes and regulates the expression of CCL2

**Fig. 1:**
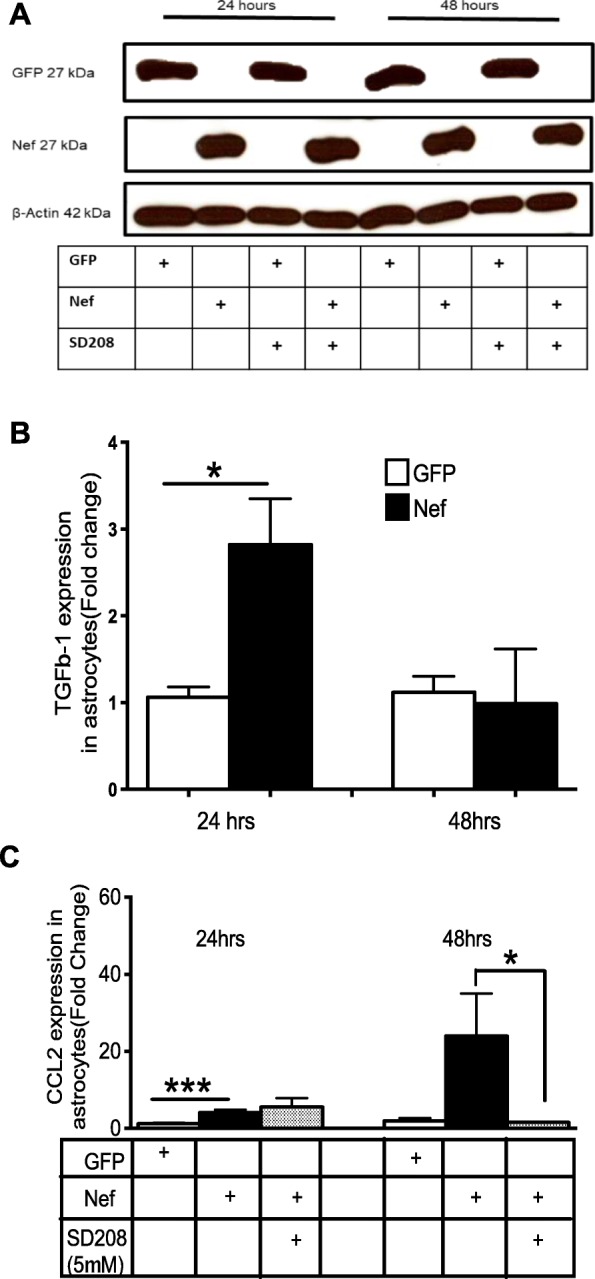
Nef-dependent upregulation of CCL2 at 48 h requires TGFβ-1 signaling. **a** GFP and Nef are expressed strongly for 48 h after transfection of rat primary astrocytes in vitro regardless of treatment with SD208, *n* = 3. **b** Quantitative PCR of TGFβ-1 expression in astrocytes expressing GFP or Nef at 24 and 48 h. GFP *n* = 10; Nef *n* = 9; *p = 0.03. **c** Quantitative PCR of CCL2 expression in astrocytes expressing GFP or Nef with or without SD208 at 24 and 48 h: GFP (white bar) *n* = 10, Nef (black bar) *n* = 8, and Nef+ SD208 (shaded bar) *n* = 3; ***p < 0.001, *p = 0.03

TGFβ-1 is rapidly produced when neuronal injury occurs [27] and has been detected in brain lesions in the astrocytes of HIV encephalitis patients [38]. TGFβ-1 also stimulates monocyte recruitment to the site of the damage by inducing the chemoattractant CCL2 [39, 40]. We previously reported that CCL2 was upregulated both in vitro and in vivo by Nef expression in astrocytes [15]. Therefore, in this investigation, we addressed the question of whether TGFβ-1 was involved in our Nef neuropathogenesis model and contributed to the CCL2 expression. Cultures of primary rat astrocytes were transfected to express the GFP (control) or Nef protein. Figure 1a shows that GFP and Nef protein expression is sustained for 48 h and that treatment with the TGFβRI inhibitor SD208 does not alter GFP or Nef expression. This result confirmed that the transfection method was efficient and suitable to proceed with experiments.

Astrocytes expressing Nef induced the expression of TGFβ-1 approximately 3-fold compared to astrocytes expressing GFP (*p* = 0.03) as shown in Fig. 1b. The increased levels of TGFβ-1 were transient and returned to baseline by 48 h. This increase in TGFβ-1 preceded an induction of CCL2, a cytokine upregulated in HAND [41, 42]. In vitro, we found the induction of CCL2 was delayed compared to TGFβ-1. The observation that TGFβ-1 upregulation precedes that of CCL2 by 24 h suggested a link to CCL2 expression. In fact, others have found that HIV trans-activator (Tat) protein induces TGFβ-1, resulting in the stimulation of SMADs and upregulating the CCL2 expression in astrocytes [43]. To test the possible role of TGFβ-1 signaling in CCL2 induction, we transfected astrocytes with Nef and treated with or without SD208 (5 μM), a competitive antagonist of TGFβRI [33], and then isolated RNA at 24 and 48 h. Figure 1c shows that Nef-expressing astrocytes have 4-fold and > 20-fold more CCL2 mRNA than GFP controls at 24 and 48 h post-transfection, respectively. Application of 5 μM SD208 had no effect on this upregulation at 24 h, but abolished the CCL2 induction completely at 48 h suggesting that the increase in TGFβ-1 induced the subsequent expression of CCL2.

### Nef increases intracellular TGFβR1 in astrocytes

**Fig. 2:**
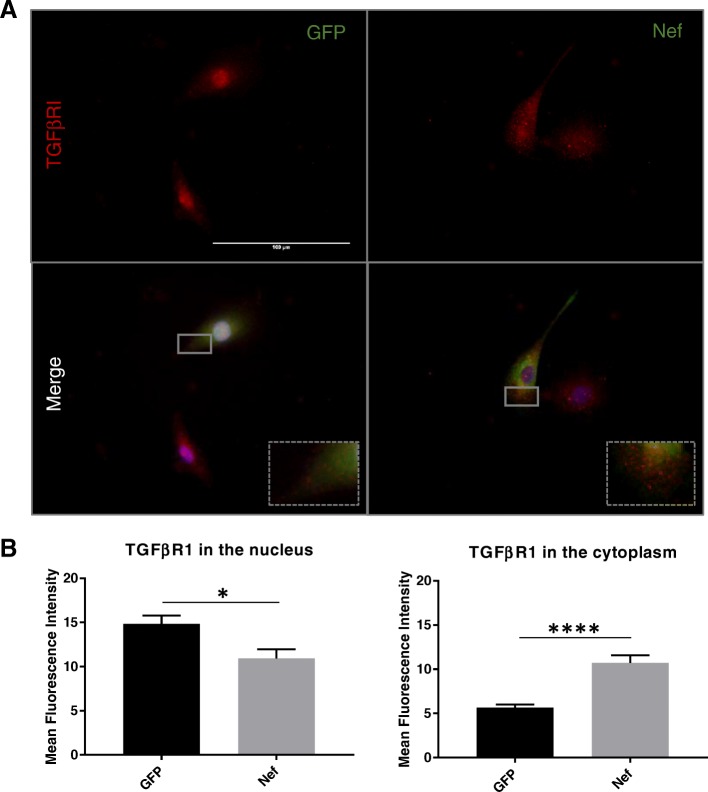
Nef increases TGFβR1 expression in astrocytes. **a** Representative images of TGFβR1 expression in astrocytes transfected with GFP or Nef (TGFβRI in red, GFP or Nef in green, DAPI in blue). Additional groups of the transfected cells were treated with SD208. Ten images per treatment from three individual experiments were obtained. **b** Quantification of mean fluorescent intensity in the nucleus (*p* = 0.02) and cytoplasm (*p* < 0.0001) of the cells. Images were analyzed with ImageJ. Scale bar is 100 μm

TGFβRI is activated by phosphorylation upon TGFβ1 binding. This event precedes phosporylation of SMAD2, complex formation with SMAD4, and nuclear translocation of the SMAD complex. Murine and rat primary astrocytes express TGFβRI (ALK5), and the reported staining patterns are similar to our results [44, 45]. Immunostaining in vitro (Fig. 2) confirms that astrocytes express the TGFβRI and found that Nef-transfected astrocytes showed stronger fluorescent punctate pattern in the cytoplasm (*p* < 0.0001) than in the nucleus (*p* = 0.02) when compared to GFP control. Other groups have suggested that the TGFβR1 staining in the nucleus can be non-specific; however, importin β1, nucleolin, and SMAD2/3 are reported to stimulate nuclear transport of the receptor in HER2 cells [46]. The nuclear staining of TGFβR1 in Fig. 2 suggests activation-induced endocytic trafficking of the TGFβR1 [47] when stimulated by Nef. Treating the cells with SD208 reduced the expression of the TGFβRI in the cytoplasm of the cells transfected with Nef and made them comparable to the control group.

### Nef does not reduce total SMAD4 in astrocytes or neurons

**Fig. 3:**
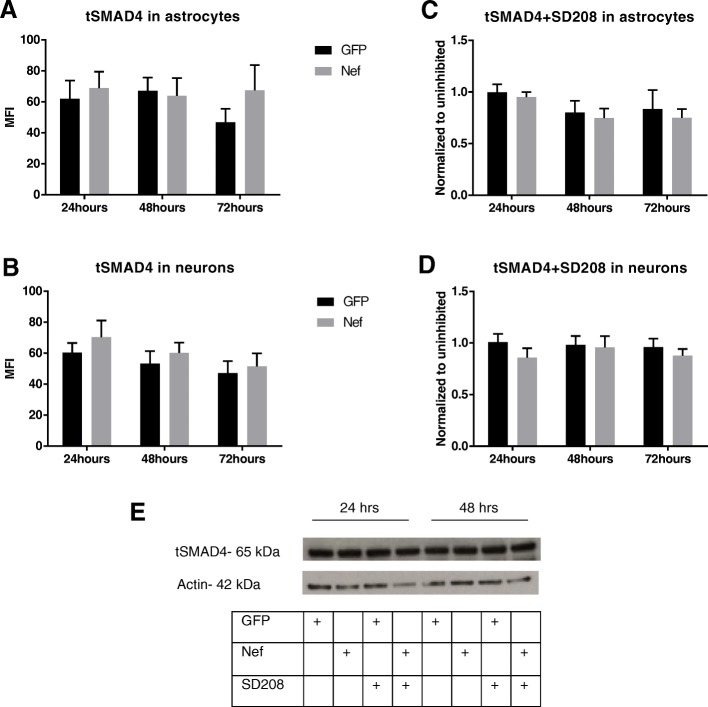
Nef does not change the amount of total SMAD4 in astrocytes and neurons. **a**–**d** Total SMAD4 expression in astrocytes and neurons show no significant difference between Nef and GFP samples; neither when treated with SD208. **e** Representative immunoblot of total SMAD4 in astrocytes expressing Nef or GFP confirms total SMAD4 protein expression

Specific SMAD2 phosphorylation is necessary for the formation of the SMAD2/4 complex in mammallian cells [48]. Interaction between activated TGFβRI, phosphorylated SMAD2, and SMAD4 can determine if the TGFβ signaling will occur via the canonical or non-canonical pathway. In vitro studies have shown that with increased time of exposure to TGFβ, expression of total SMAD4, and total SMAD2 in a rat pituitary cell line remain the same, while the phosphorylation of SMAD2 increases with treatment [49]. This is consistent with our findings. We quantified the expression of total SMAD4 in astrocytes (transfected with Nef or GFP) and neurons that were co-cultured for up to 3 days. Groups treated with SD208 were included. There was no significant difference in total SMAD4 expression in Nef samples when compared to GFP controls, in neither samples treated with SD208 (Fig. 3a–d). Figure 3e shows a representative immunoblot for total SMAD4 in astrocytes which confirms unchanged levels of total SMAD4 by Nef.

### Inhibiting TGFβRI rescues learning in Nef-treated rats

**Fig. 4:**
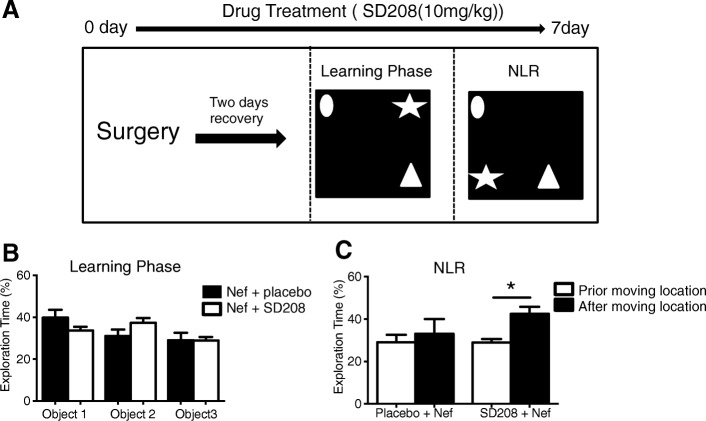
SD208 rescues the spatial learning impairment in rats treated with Nef. **a** A schematic of behavioral model. **b** Average exploration time of rats from two groups (Nef + placebo, black bar, *n* = 7; Nef + SD208, white bar, *n* = 13) for each object during learning phase; **c** percent exploration time for the test object prior to (open bars) and after (closed bars) moving the location. All error bars represent standard error. *p < 0.05

Expression of Nef by astrocytes infused into the dentate gyrus impairs spatial learning in rats [15]. CCL2 expression is increased at the site of injection in vivo and correlates with an increase in CD163-positive cells [15], a marker for the macrophage scavenger receptor [50], suggesting the CCL2 was active in chemotaxis. The timing of expression we found in vitro shown in Fig. 1 suggests that TGFβ-1 could be involved in the Nef-induced CCL2 upregulation seen in vivo that was found in animals with learning impairment along with infiltration of macrophages to the hippocampus [15]. To test involvement of TGF in the observed inflammation and learning impairment, we infused astrocytes expressing Nef into the right hemisphere of the hippocampus and administered SD208 (10 mg/kg) orally to antagonize the effects of TGFβ-1 for seven consecutive days. The rats were then tested for memory (Fig. 4a). Results in Fig. 4b show that the rats treated with Nef with or without the TGFβR1 inhibitor exhibited no object preference (Nef + placebo group = 7, Nef + SD208 = 13, *p* > 0.05) in the learning phase. Consistent with our previous results, Nef-treated rats that received placebo showed memory impairment in the novel location recognition (NLR) task, since they showed no preference to explore the moved object. In contrast, rats treated with SD208 showed good spatial memory in the NLR task and spent more time exploring the moved object (Fig. 4c, *p* = 0.01). This shows that the learning impairment in our Nef neuropathogenesis model requires signaling via TGFβ-1.

### SD208 does not alter motor performance, weight gain, or anxiety levels of Nef-treated rats

**Fig. 5:**
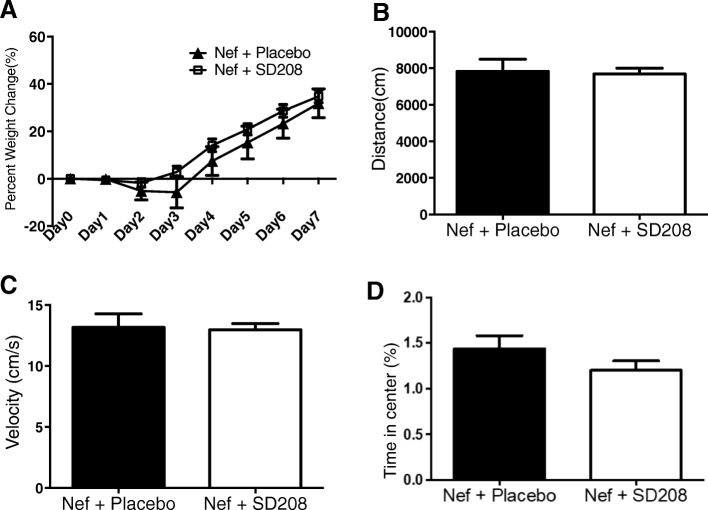
SD208 does not affect weight, motor performance, or anxiety levels. **a** Percent of weight change during the administration of the treatment. **b** Distance traveled during the testing phase. **c** Velocity of the rats during the learning phase. **d** Time spent in the center of the area during the testing phase (an indicator of anxiety). *p* > 0.05, *n* = 7;13

Previous studies showed that learning impairment can be caused by stress or anxiety [51, 52]. Our model has already been validated to demonstrate that infusion of astrocytes expressing an HIV protein does not cause changes in thigmotaxis nor locomotor activity, indicating that neither anxiety nor motor disturbances were the cause of the learning impairment [15, 34]. Similarly, we found that neither SD208 nor the methylcellulose vehicle cause changes in weight, distance traveled, velocity, or anxiety levels, thus eliminating these variables as possible explanations for differences in learning after SD208 treatment. Figure 5a shows that there was no difference in weight gain between the SD208-treated group and the placebo group. During the testing phase, we measured distance traveled (Fig. 5b), velocity (Fig. 5c), and time spent in the center as an indicator of anxiety (Fig. 5d) and found no differences between the groups (Nef + placebo, *n* = 7, and Nef + SD208, *n* = 13). Therefore, the TGFβ-1 inhibitor does not affect the mobility, weight, or anxiety levels of the rats suggesting that the effect is specific to learning.

### TGFβRI antagonist inhibits CCL2 expression in vivo

**Fig. 6:**
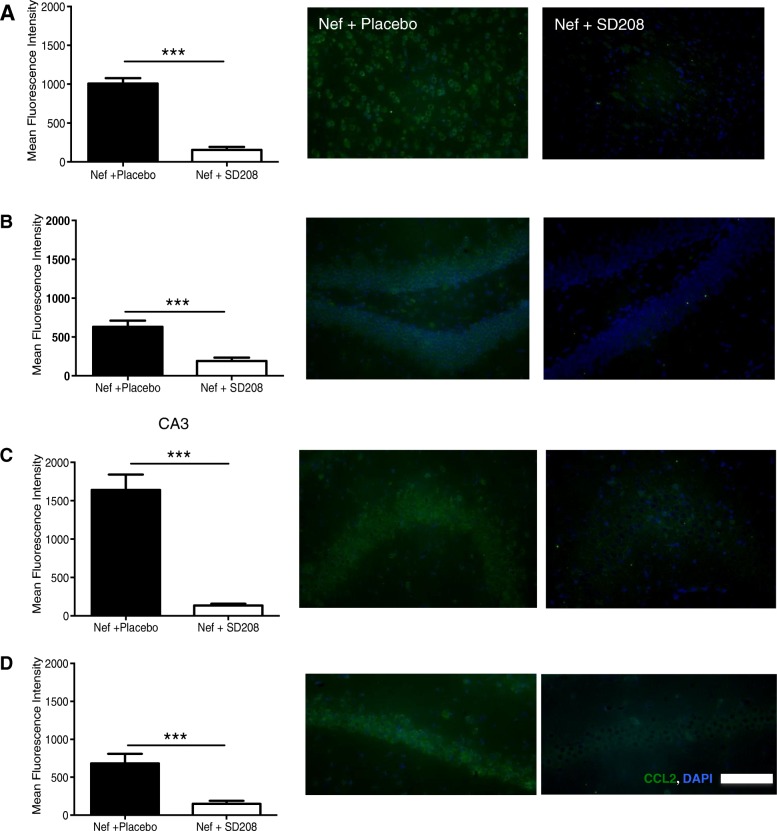
Blocking TGFβR1 with SD208 decreases CCL2 expression in rats infused with astrocytes expressing Nef. Mean fluorescence intensity and representative rat brain tissue sections immunostained for macrophage chemoattractant marker CCL2 (green): cortex (**a**), dentate gyrus (**b**), CA3 (**c**), and CA1 (**d**). ***p < 0.0001. Scale bar is 100 μm

Our work and that of others show that Nef induces CCL2 expression in astroctyes in vitro and in vivo [15, 53]. Studies of tissues other than brain show that TGFβ-1 has chemoattractive function and that it can regulate CCL2 [54, 55]. Interestingly, treating with TGFβ-1 did not increase CCL2 gene expression and led to improved function in a microglia-mediated inflammation study of intracerebral hemorrhage [56]. On the other hand, in a model of autoimmune encephalomyelitis, astrocytes produced TGFβ along with increased inflammation and activation of the TGFβ signaling pathway in neurons, with all effects prevented by inhibition of TGFβR1 [24]. In Fig. 1c, we demonstrate that Nef-induced CCL2 expression can be prevented by a TGFβRI antagonist in vitro. To test whether TGFβ contributes to Nef-induced CCL2 expression in the brain, animals were implanted with astrocytes expressing Nef. Knowing beforehand that Nef increased CCL2 expression in rat brain when compared to GFP controls guided us to study if Nef-treated groups show differences in CCL2 expression when the placebo or TGFβRI antagonist was administered. One group was given placebo, and the other group was given SD208 (10 mg/kg) orally for seven consecutive days. Animals were euthanized, and CCL2 expression was measured by immunofluorescence in hippocampal sections. Our data shows that animals exposed to astrocytes expressing Nef without SD208 (placebo group) have an increase in CCL2 expression in the brain including cortex, dentate gyrus, CA3, and CA1 which is consistent with our previously reported results. SD208 abrogated this increase in CCL2 expression in these brain regions (Fig. 6). This suggests that the TGFβ-1 signaling pathway induces the recruitment of macrophages into the brain through induction of CCL2 in the presence of Nef.

It is important to note that the surgery performed in the rats is an intracranial infusion which mechanically can produce inflammation independent of the effect of Nef. We have previously described our results using Nef and GFP as control to account for this effect. Yet, studies in rat brains have reported constitutive expression of CCL2 not only in astrocytes but also in neurons located at the cerebral cortex and hippocampus [57].

### Blocking TGFβRI reduces phospho-SMAD-2 in rats infused with astrocytes expressing Nef

**Fig. 7:**
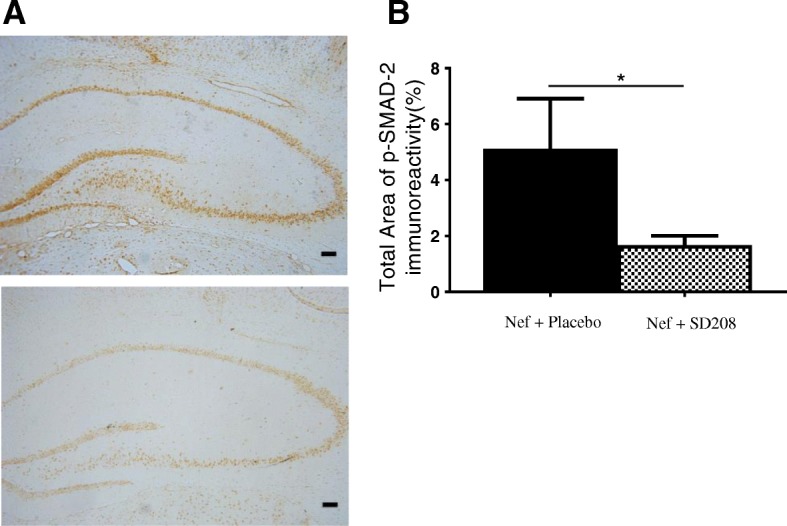
Blocking TGFβR1 with SD208 decreases phospho-SMAD2 in rats infused with astrocytes expressing Nef. **a** Representative tissue sections immunostained for phospho-SMAD2; Nef+placebo (upper panel) and Nef+SD208 (lower panel). **b** Densitometric analysis of the hippocampus was used to quantify SMAD2 phosphorylation (right hemisphere: *p < 0.03). Nef+placebo (*n* = 5) and Nef+SD208 (*n* = 6). Scale bar is 100 μm

Inflammatory mediators can induce astrocytes to release TGFβ-1 which can cause SMAD2 phosphorylation in hippocampal neurons and result in neuronal injury [58]. In addition, TGFβ-1 can increase glutamate-evoked currents by the upregulation of glutamate receptors in the hippocampus [29]. Our observation that SD208 treatment prevented the Nef-associated spatial learning impairment suggests that Nef requires TGFβ signaling for neurotoxicity. Therefore, we used immunohistochemistry to assess SMAD2 phosphorylation, a downstream component of the TGFβ signaling pathway. Nef-treated rats that received the TGFβRI inhibitor showed an approximate 3-fold decrease in phospho-SMAD2 immunoreactivity in both hippocampal hemispheres suggesting that the inhibitor acts in the hippocampal neurons (right hemisphere—site of injection: *p* < 0.01; Nef + placebo group *n* = 4 and Nef + SD208 *n* = 6; Fig. 7). The bilateral effect was expected since the SD208 was administered orally. The restoration of learning in the Nef + SD208 group suggests that the impairment in learning caused by Nef depends on TGFβ signaling via SMAD2.

### Inhibiting TGFβRI decreases the CD163-positive cells at the site of the injection

Previously our laboratory showed that astrocytic Nef expression in the hippocampus increases infiltration of macrophages at the site of the injection when compared to GFP controls [15]. The finding that Nef, through TGFβ-1, induces CCL2 in vitro (Fig. 1c) and in vivo (Fig. 6) suggests a mechanism by which these macrophages target the site of Nef production.

**Fig. 8:**
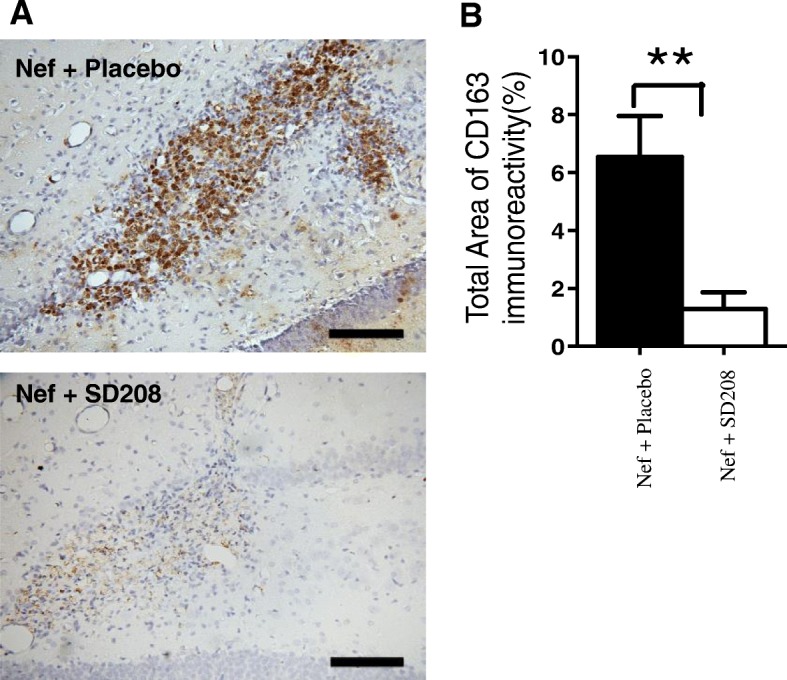
Blocking TGFβR1 with SD208 decreases CD163 expression in rats infused with astrocytes expressing Nef. **a** Representative tissue sections immunostained for macrophage marker, CD163. Nef+placebo (upper panel), *n* = 4 and Nef+SD208 (lower panel), *n* = 5. **b** Densitometric analysis was used to quantify the intensity of CD163 staining, **p < 0.01. Scale bar 100 μM

Therefore, we examined whether inhibition of TGFβRI decreases the immunoreactivity against CD163 at the site of the injection of Nef-expressing astrocytes. Figure 8a shows that the rats treated with Nef + SD208 exhibit decreased expression of CD163-positive cells at the site of injection. Densitometric analysis in Fig. 8b indicates that SD208 reduces infiltration by approximately 4-fold (*p* = 0.01) suggesting that TGFβ-1 signaling enhances the infiltration of macrophages in response to Nef expression in astrocytes.

### Inhibiting TGFβRI decreases the GFAP expression in rats infused with astrocytes expressing Nef

Astrocytosis is one of the hallmarks of HIV neuropathology [59]. The glial fibrillary acidic protein (GFAP), which is used as a marker for reactive astrocytes, increases during HIV-1 infection [60] and has helped identify astrocytes as a significant viral reservoir in the brain [61]. The adequate function of astrocytes is relevant to maintain a suitable environment for neurons and other glial cells. Other researchers have demonstrated that astrocytes infected with HIV have metabolic interactions with neurons that can result in dysregulation of calcium flux [62], limited glutamate reuptake [63], and altered blood brain barrier integrity [64].

**Fig. 9:**
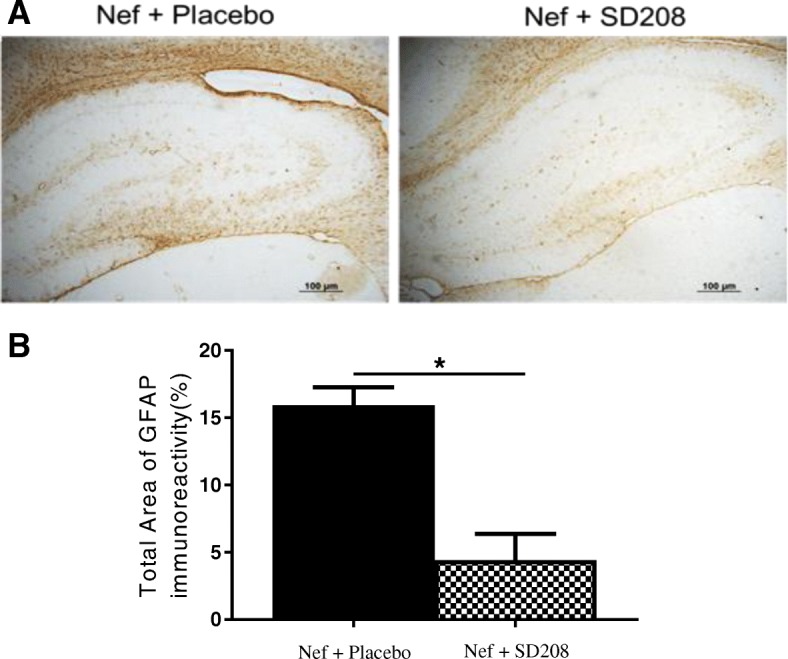
Blocking TGFβR1 with SD208 decreases GFAP expression in rats infused with astrocytes expressing Nef. **a** Representative tissue sections immunostained for astrocyte activation marker, GFAP. Nef+placebo (left panel), *n* = 5, and Nef+SD208 (right panel), *n* = 3. **b** Densitometric analysis was used to quantify the intensity of GFAP staining, *p < 0.03. Scale bar 100 μM

Treating astrocytes with TGFβ-1 increases GFAP mRNA and protein [65]. In ALS, increased levels of CSF TGFβ-1 significantly correlated with duration of disease and in a mouse model was found to interfere with neuroprotective function of immune cells [66, 67]. Here, we quantified the expression of GFAP on Nef-treated animals. Inhibiting the TGFβR1 leads to decreased immunoreactivity against GFAP. Figure 9a shows that the rats treated with Nef + SD208 exhibit decreased expression of GFAP. Densitometric analysis in Fig. 9b indicates that SD208 significantly reduces astrocytosis (*p* = 0.04) suggesting that TGFβ-1 signaling enhances the astrocytes activation in response to Nef expression in astrocytes.

## Discussion

HIV-associated neurocognitive disorders (HAND) are clinically important comorbidities presented in about half of HIV+ individuals, even those receiving cART. This represents a problem, not only because memory is impaired but also because it can lead to the interruption of therapy and eventually increased viral load. Our focus is the role of Nef in HAND, which mechanism is not well elucidated. HIV-1 Nef is an early viral protein that can be produced by astrocytes even in the absence of active viral replication, and causes the increase of inflammatory cytokines and chemokines including IP-10, IL-6, and IL-8 [6, 68]. Previously, we demonstrated that Nef increases the expression of macrophage chemokine CCL2 and macrophage marker CD163 which correlate with cognitive deficits observed in our animal model when compared to controls. The goal of this study is to extend those findings by assessing if TGFβ1 signaling is an important player for the inflammation and learning impairment. We found an early, transient increase in TGFβ in cultures of primary rat astrocytes expressing Nef followed by increased expression of CCL2. Based on reports that TGFβ could stimulate chemotaxis through CCL2 [39, 40], we tested the effect of a competitive antagonist of TGFβRI, SD208, on CCL2 expression in rats infused with Nef-transfected astrocytes and found a substantial block. We also found that treatment with SD208 restored spatial learning in rats despite Nef expression in the hippocampus. The rescue of learning in the Nef-treated animals by a competitive antagonist of TGFβRI was associated with decreased perivascular macrophage infiltration and astrogliosis in the hippocampus, assessed immunohistochemically using CD163 and GFAP, respectively. In addition, primary astrocytes and brain tissues from rats showed a redistribution of the TGFβRI and decreased expression of CCL2 and phosphorylated SMAD2. Our data suggest that TGFβ1 signaling is key to the learning impairment in our model of astrocytic Nef neuropathology.

Although the entire hippocampus is involved in memory, the right side of the hippocampus is noted for a major role in episodic and spatial memory in animal models as well as in humans [69, 70]. Using our established model of Nef-induced learning impairment that targets the right hippocampus, we extended our study to TGFβ-1 signaling in the neuropathology caused by Nef with the use of two groups: Nef + placebo and Nef + SD208. Young rats were selected to avoid normal aging as a contributor to learning impairment. Previously, we showed that the HIV-1 neurotoxic proteins Nef [15] and Vpr [34] cause spatial learning impairment when expressed in the hippocampus. A study from a different group of researchers showed that hippocampal implantation of astrocytes expressing another HIV neurotoxic factor, Tat protein, also caused glial activation and neurotoxicity in vivo; however, they did not test learning [37]. Altogether, these studies establish that infusion of astrocytes into the hippocampus is a model to study the effect of specific viral proteins in the learning deficits presented in HAND and that local production of a single neurotoxic HIV protein can cause damage and behavioral effects consistent with some impairment found in HAND.

Several aspects of our model resemble human HIV infection and HAND. In the case of HIV-infected individuals with neurocognitive impairment, there is evidence of increased CCL2 in their CSF and central nervous system (CNS) [71, 72]. HIV infection targets discrete cells in the brain, predominantly microglia, macrophages, and astroctyes. Astrocytes are an atypical HIV target, recognized for a non-productive infection, but also linked to worse neurological disorders with increasing occurrence of infection [61, 73–76]. Infected cells produce and secrete CCL2. This increase in CCL2 has also been linked to the interaction of viral proteins with the TGFβ signaling pathway [43]. Early viral proteins, particularly Nef, are produced by astrocytes in HIV-infected persons [7, 9]. Recent studies have pointed to continued production of Nef even in individuals with undetectable plasma virus [77], suggesting a role for early viral proteins in ongoing morbidities, such as HAND, in virally suppressed individuals. Astrocytes can produce Nef and also increase the secretion of TGFβ which can be detrimental for neurons. Normally, astrocytes support neurons by releasing growth factors that help to maintain homeostasis in the brain. However, in neurodegenerative diseases such as HAND, astrocytes change their normal morphology to a star-shaped morphology reflecting reactive astrogliosis. This change promotes the activation of more astrocytes and the release of proinflammatory mediators considered important to HAND pathology such as CCL2. Our results agree with studies showing that overproduction of TGFβ-1 promotes astrogliosis [29, 58, 78] as well as our earlier work and that of others indicating that Nef induces expression of a potent monocyte chemoattractant, CCL2, by astrocytes [15, 16].

Since TGFβ-1 displays a diversity of cell functions including chemoattractant properties [54, 79, 80], we wanted to confirm the role of this signaling pathway in Nef-mediated inflammation. Reported concentrations of the TGFβR1 competitive inhibitor SD208 used for in vitro studies usually vary between 1 μM and 10 μM and up to 50 mg/kg by gavage for in vivo studies with mice [81–83]; our work falls within these ranges. When we added SD208 (5 μM in vitro; 10 mg/kg in vivo), we found inflammation caused by Nef was reduced. In vivo, we also demonstrated that blocking TGFβ signaling with SD208, confirmed by a reduction in phospho-SMAD2, decreased CCL2 production. This observation is similar to that of a mouse model of interstitial lung disease showing that inhibition of TGFβ signaling by using a TGFβRI inhibitor decreased mRNA of chemoattractant cytokines including CCL2 [84]. Levels of CCL2 in CSF in humans correlate with the learning impairment in some HIV-positive individuals [85] presumably due to the role of this powerful chemoattractant in drawing monocytes to the brain. In a mouse model of traumatic brain injury, a deficiency in CCR2 led to a reduction of macrophages in the brain, a better outcome in neuronal survival, and better spatial learning and memory [86]. Also, with the use of a selective antagonist for CCR2 (the CCL2 receptor) in a traumatic brain injury model, accumulation of macrophages in the hippocampus was reduced as were cognitive impairment and inflammation [87]. Similarly, cognitive impairments caused by cranial irradiation in mice were prevented by a CCR2 knockdown, which suggests that this receptor is a mediator of neuronal damage following injury and inflammation [88]. These studies show the relevance of CCL2 and its receptor CCR2 in neuropathologies. We find significant macrophage infiltration in our Nef-treated rats that is prevented by SD208 treatment, in parallel to a decrease in CCL2 production. Consistent with the aforementioned studies, interference with CCL2 and macrophage infiltration correlated with restoration of learning in our Nef animal model.

A more thorough characterization of mechanism is warranted to better understand the link between TGFβ and CCL2, particularly as our data suggest there is some activity that remains in the presence of SD208. Although such studies extend beyond the scope of this work, we included the in vitro experiments to demonstrate sufficiency of the repression by SD208 in the inflammation (observed by infiltration of CD163-positive cells in the hippocampus), but herein we did not intend to prove mechanism. Nevertheless, other researchers have described how the TGFβ pathway regulates CCL2 expression resulting in enhanced or limited inflammation in the brain and other organs [89–91]. TGFβ has macrophage chemoattractant properties, and regulatory loops between TGFβ and CCL2 (MCP1) have been identified, involving the ERK and AKT pathways [55, 92]. To investigate the role of TGFβ in these and other studies, receptor knockdown and inhibitors, such as SB431542, have been used.

TGFβ signaling activates SMAD family proteins, starting with phosphorylation of SMAD2, and induces nuclear translocation as part of a complex with SMAD3 and SMAD4. This complex directly regulates transcription of TGFβ responsive genes [93, 94]. Specifically, TGFβR1 and SMAD4 play important roles in the control of neuronal morphogenesis and have shown to prevent elongation of neurons [95]. These dysregulations can result in compromised brain function, and mutations in TGFβRI and SMAD4 can result in cognitive impairment among other mental disorders [96, 97]. TGFβRI controls neuron maturation in the adult hippocampus [98]. Furthermore, TGFβ1 can activate two types of receptors, ALK5 or ALK1, which can be neurodegenerative or neuroprotective, respectively [28].

We further investigated the activity of TGFβ in our Nef-treated rats by showing SMAD2 activation in the hippocampus that was prevented by SD208. Along with SMAD2 activation, we observed availability of total SMAD4 and increased cytoplasmic expression of the receptor with Nef when compared to control. TGFβ receptors are known to be found intracellularly and, typically in response to a stimuli, receptors translocate to the plasma membrane to facilitate TGFβ pathway activation [99]. For example, exposure of fibroblasts and epithelial cells to high levels of glucose increases the TGFβ receptors in the cell membrane and there is activation of the TGFβ ligand resulting in cell hypertrophy [100]. In a study specifically using HER2-transformed cells, nuclear translocation of TGFβR1 was observed as shown with immunoprecipitation and immunofluorescence assays. The nuclear translocation of TGFβR1 binding proteins indicated a molecular function in RNA posttranscriptional modifications. Furthermore, nuclear translocation induction with a TGFβ ligand or a constitutively induced TGFβR1 proved to be context dependent. The nuclear proteins importin β1 and nucleolin along with transient interactions with SMAD2/3 were required for the TGFβR1 nuclear translocation [46]. A different study shows that TGFβ induces the nuclear localization of TGFβR1 in various cancer cells by a Lys-63-dependent polyubiquination. The C-terminal domain of the TGFβR1 had the most accumulation in the nucleus whereas the N-terminal was most abundant in the cytoplasm. This nuclear accumulation was also observed in human tumors [101]. Other studies have demonstrated an important relationship between TGFβR1, also known as ALK5, and endocytic structures [102]. Relocalization of the receptors is dependent on ligand binding and relies on the ESCRT pathways [103]. Interestingly, Nef uses the ESCRT pathway to downregulate CD4 [104] and we have shown that Nef induces TGFβ gene.

Thus, a complex relationship remains to be further studied; however, our findings support Nef perturbation of TGFβ signaling at the cellular level as a component of the mechanism for Nef neurotoxicity and its alleviation by SD208 in vivo. Our results suggest that a dysregulation of TGFβ signaling in the hippocampus may produce learning impairment and that Nef is an HIV-1 neurotoxin capable of initiating neuropathology by this route. We show that hippocampal inflammation increases with astrocytic Nef expression and that the canonical TGFβ signaling has a role in the cascade of events leading to Nef-mediated neurotoxicity and learning impairment. In this regard, our future studies include the characterization of the TGFβ canonical pathway to better understand its role in Nef-mediated inflammation.

Association of Nef with HIV neuropathology has been known since relatively early in the epidemic. Tissues from children who had AIDS encephalopathy present significant Nef mRNA and protein expression specifically by astrocytes in contrast with structural proteins which are mainly found in multinucleated giant cells [105]. In a mouse model of HIV-1 encephalitis, the animals showed impaired synaptic function and long-term potentiation when compared to sham animals, which are similar to the pathophysiology in HIV-1-associated dementia [106]. These results suggests that Nef induces neurotoxicity by the release of inflammatory cytokines and by regulating synaptic plasticity. Therefore, bringing the attention to the role of the TGFβ pathway on neuron fate and how this can be modulated by astrocytic Nef is relevant, particularly in light of findings suggesting Nef is expressed in virally suppressed individuals [77]. TGFβ signaling can be neurotoxic by promoting overexpression of NMDA receptor subunits, increasing glutamatergic currents, and causing an increase of calcium influx into hippocampal neurons [107, 108]. TGFβ also can increase neuroinflammation by downregulating TIMP-1 in astrocytes, and altered balance between MMPs and TIMPs are associated with HAD [109, 110]. Increased levels of calcium may produce oxidative stress resulting in neuron death. Other studies have also found a correlation between increased intracellular calcium levels induced by substance P and high expression of CD163 in HIV infection [111]. Furthermore, blocking TGFβR1 with the use of SD208 in a rat model of germinal matrix hemorrhage delayed the learning impairment and astrocyte activation in the hippocampus [33].

Herein, we suggest a molecular mechanism, the TGFβ signaling pathway, as a route to be further investigated and targeted in order to better understand and treat HAND. This cascade regulates many of the inflammatory cytokines that have been identified by other groups as contributors of the neuroinflammation observed in HIV+ individuals. It has also been well studied in other neuropathologies, demonstrating its role in brain development and disease. Therefore, the data presented in this study provides bases to consider TGFβ signaling as a regulator of the Nef-mediated neurotoxicity and neuropathology.

## Conclusion

**Fig. 10:**
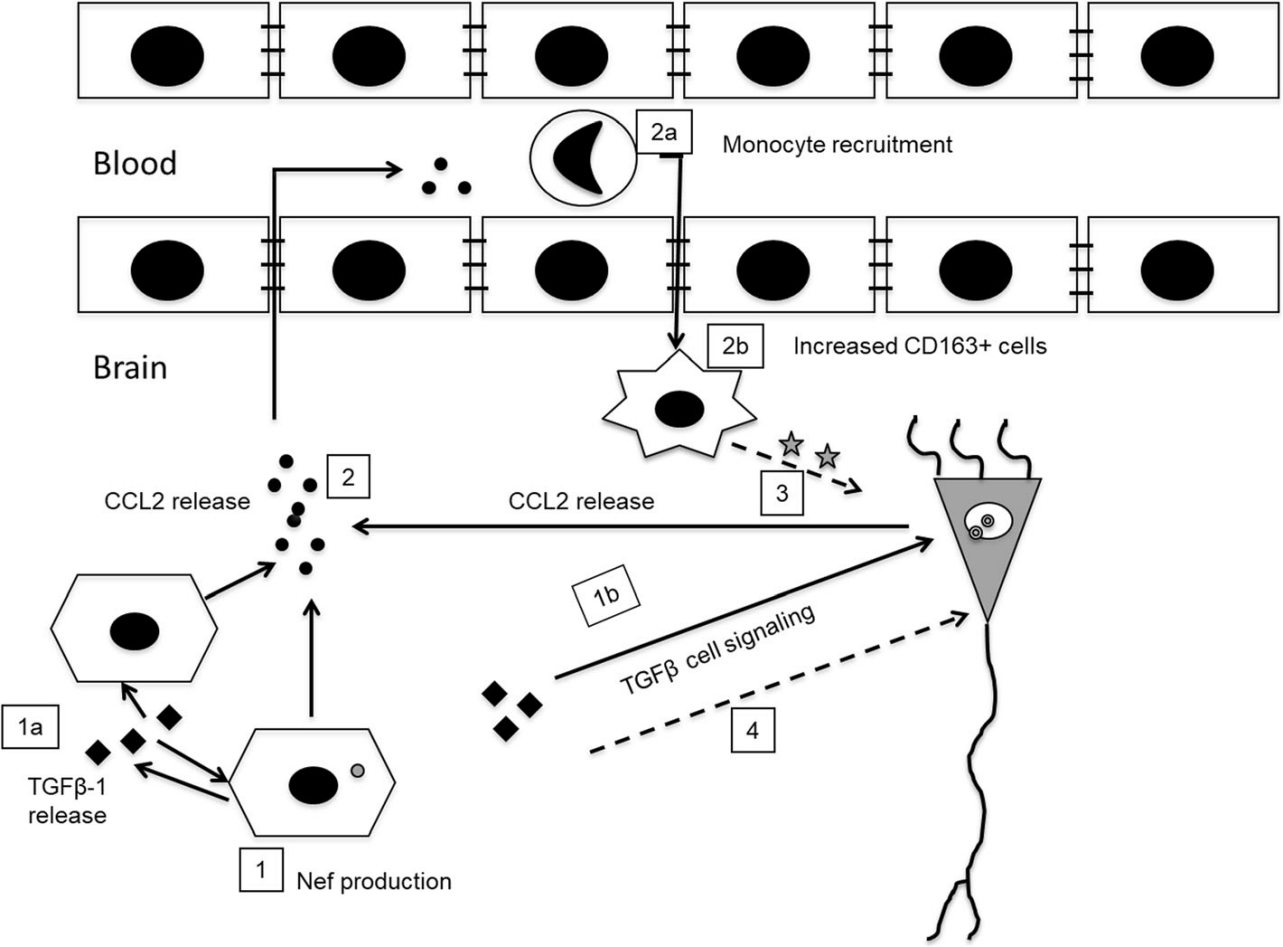
Proposed model of the involvement of HIV-1 Nef and TGFβ-1 in neuroinflammation and learning impairment. Astrocytes expressing Nef (1) release TGFβ-1 acting in paracrine and autocrine fashion (1a) to stimulate the TGFβR of astrocytes and adjacent neurons (1b). This activates SMAD signaling and induces CCL2 (2), a potent chemoattractant that results in mononuclear cell infiltration (2a) and subsequent differentiation into macrophages expressing the scavenger receptor CD163 (2b). The consequent inflammation results in astrogliosis and damage to hippocampal neurons (3) as an underlying cause of the spatial learning impairment. TGFβ-1 also can drive direct neurotoxicity by increased glutamatergic activity and intracellular calcium (4). Solid lines indicate supported by data in this study and in the literature. Dashed lines are conclusions drawn from previous studies and work of others

Our results support the model where astrocytes expressing Nef promote the expression of TGFβ-1 which acts in the hippocampus to stimulate SMAD-2 phosphorylation resulting in CCL2, CD163, and GFAP upregulation. CCL2 attracts perivascular macrophages to migrate into the brain, causing an increase in inflammation that drives neurotoxicity leading to learning impairment (Fig. 10). Our findings suggest that inhibitors of the TGFβ signaling pathway may be useful for the prevention of cognitive impairment seen in patients with HAND.

## Data Availability

The datasets used and/or analyzed during the current study are available from the corresponding author on reasonable request.

## Abbreviations

ANI: Asymptomatic neurocognitive impairment
ANOVA: Analysis of variance
cART: Combination antiretroviral therapy
CCL2: CC chemokine family ligand 2
CD163: Cluster of differentiation 163
CMV: Cytomegalovirus
CNS: Central nervous system
CXCL10/IP-10: CXC chemokine family ligand 10/interferon gamma-induced protein 10
DAB: Diaminobenzidine
EDTA: Ethylene diamine tetra acetic acid
GFAP: Glial fibrillary acidic protein
GFP: Green fluorescent protein
HAD: HIV-associated dementia
HAND: HIV-associated neurocognitive disorders
HIV: Human immunodeficiency virus
HRP: Horse radish peroxidase
IHC: Immunohistochemistry
IL-6: Interleukin 6
IL-8: Interleukin 8
MND: Mild neurocognitive disorder
Nef: Negative factor
NLR: Novel location recognition
PBS: Phosphate-buffered saline
qPCR: Quantitative polymerase chain reaction
SMAD: Mothers against decapentaplegic homolog
Tat: Trans-activator of transcription
TGFβ-1: Transforming growth factor beta 1
TGFβRI: Transforming growth factor beta receptor 1
Vpr: Viral protein R
